# Co-Lateral Effect of Octenidine, Chlorhexidine and Colistin Selective Pressures on Four Enterobacterial Species: A Comparative Genomic Analysis

**DOI:** 10.3390/antibiotics11010050

**Published:** 2021-12-31

**Authors:** Mathilde Lescat, Mélanie Magnan, Sonia Kenmoe, Patrice Nordmann, Laurent Poirel

**Affiliations:** 1 Emerging Antibiotic Resistance Unit, Medical and Molecular Microbiology, Faculty of Science and Medicine, University of Fribourg, 1700 Fribourg, Switzerland; mathilde.lescat@inserm.fr (M.L.); patrice.nordmann@unifr.ch (P.N.); 2 INSERM European Unit (LEA, IAME), 75870 Paris, France; 3 Université Sorbonne Paris Nord, 93000 Bobigny, France; melanie.magnan@inserm.fr; 4 AP-HP, Hôpitaux Universitaires Paris Seine Saint-Denis, 93000 Bobigny, France; sonia.kenmoe@inserm.fr; 5 Swiss National Reference Center for Emerging Antibiotic Resistance (NARA), University of Fribourg, 1700 Fribourg, Switzerland; 6 Medical and Molecular Microbiology Unit, Department of Medicine, Faculty of Science, University of Fribourg, Chemin du Musée 18, 1700 Fribourg, Switzerland

**Keywords:** colistin, resistance, chlorhexidine, octenidine, *Enterobacterales*

## Abstract

Bacterial adaptation to antiseptic selective pressure might be associated with decreased susceptibility to antibiotics. In Gram-negative bacteria, some correlations between reduced susceptibility to chlorhexidine (CHX) and polymyxins have been recently evidenced in *Klebsiella pneumoniae*. In the present study, four isolates belonging to distinct enterobacterial species, namely *K. pneumoniae*, *Escherichia coli*, *Klebsiella oxytoca* and *Enterobacter cloacae*, were submitted to in-vitro selective adaptation to two antiseptics, namely CHX and octenidine (OCT), and to the antibiotic colistin (COL). Using COL as selective agent, mutants showing high MICs for that molecule were recovered for *E. cloacae*, *K. pneumoniae* and *K. oxytoca*, exhibiting a moderate decreased susceptibility to CHX, whereas OCT susceptibility remained unchanged. Using CHX as selective agent, mutants with high MICs for that molecule were recovered for all four species, with a cross-resistance observed for COL, while OCT susceptibility remained unaffected. Finally, selection of mutants using OCT as selective molecule allowed recovery of *K. pneumoniae*, *K. oxytoca* and *E. cloacae* strains showing only slightly increased MICs for that molecule, without any cross-elevated MICs for the two other molecules tested. No *E. coli* mutant with reduced susceptibility to OCT could be obtained. It was therefore demonstrated that in-vitro mutants with decreased susceptibility to CHX and COL may be selected in *E. coli*, *K. pneumoniae*, *K. oxytoca* and *E. cloacae*, showing cross-decreased susceptibility to COL and CHX, but no significant impact on OCT efficacy. On the other hand, mutants were difficult to obtain with OCT, being obtained for *K. pneumoniae* and *E. cloacae* only, showing only very limited decreased susceptibility in those cases, and with no cross effect on other molecules. Whole genome sequencing enabled deciphering of the molecular basis of adaptation of these isolates under the respective selective pressures, with efflux pumps or lipopolysaccharide biosynthesis being the main mechanisms of adaptation.

## 1. Introduction

Colistin (COL) is a last-resort antibiotic for treating infections caused by multidrug-resistant (MDR) Gram-negatives, and particularly carbapenemase-producing isolates [1]. Indeed, while all other clinically-available therapeutic drugs might be inefficient against these MDR bacteria, COL still shows a high rate of efficiency, with a limited number of resistant isolates identified, except in some outbreak contexts (e.g., COL-resistant carbapenemase-producing *Klebsiella pneumoniae* in Italy or Serbia [2,3]). Nevertheless, due to an increasing use of COL in recent years, an emergence of COL-resistant isolates has been observed, particularly in *K. pneumoniae*, but also in other enterobacterial species. This phenomenon is mainly observed in hospital settings, with a very low rate of COL-resistant isolates being observed in the community [4].

In general, prevention strategies within the hospital are a key element in infection control. Use of antiseptic molecules contributes significantly to the control of dissemination of methicillin-resistant *Staphylococcus aureus* (MRSA), but also to control of (multidrug resistant) Gram-negative bacteria on skin, wounds and mucous membranes [5]. In that context, use of chlorhexidine (CHX) is recommended and CHX is being heavily used for prevention and for decolonization strategies [6]. Likewise, octenidine (OCT) is another antiseptic molecule that shows excellent efficacy in eradicating MDR bacteria [7,8,9,10]. We recently performed a study evaluating the efficacy of OCT against MDR Gram negatives (including *E. coli*, *K. pneumoniae*, *E. cloacae*, *Acinetobacter baumannii* and *Pseudomonas aeruginosa*) [11]. These clinically-relevant Gram-negative pathogens were chosen for their multidrug resistance phenotype, including resistance to ß-lactams, aminoglycosides, and fluoroquinolones. OCT activity was proven to be extremely efficient against these MDR bacteria at clinically-relevant concentrations [11].

A recent study showed that in-vitro selective adaptation of *K. pneumoniae* clinical isolates to CHX may select cross-resistance to COL [12]. Acquired resistance to CHX occurred through mutations in the *phoPQ* two-component regulatory genes (mainly in the *pmrK* gene), and in the *smvR* repressor gene adjacent to the major facilitator superfamily efflux pump gene *smvA.* Such mutations had significant co-lateral effects on susceptibility to COL, with MICs increasing from 2 to 4 µg/mL (original strain) to >64 µg/mL for most of the mutant strains. By contrast, no significant change was observed for OCT susceptibility, highlighting the very limited impact of those mutations selected on CHX on the efficacy of that other antiseptic. In that same study, it was shown that the opposite strategy—to select COL-resistant mutants—had no significant impact on CHX susceptibility, despite similar mutations in the *phoPQ* two-component regulatory genes being identified. The study thus showed that the latter mutations were necessary but not sufficient to confer resistance to CHX [12]. Of note, the latter study only focused on the *K. pneumoniae* species, and only adaptation on CHX was considered in the design of the study.

Using strains belonging to four *Enterobacterales* species, namely *K. pneumoniae*, *K. oxytoca*, *E. coli*, and *E. cloacae*, the adaptation to CHX, COL, and OCT was examined. Our working hypothesis was that use of the antibiotic colistin that acts on the outer membrane of Gram-negative cells might have some impact on susceptibility to antiseptics, and vice versa. The objectives of our study were therefore the following: (i) to evaluate whether mutants could be selected in-vitro with CHX, COL, and OCT for the aforementioned enterobacterial species, (ii) to further evaluate the extent of the inter-relation between reduced susceptibility to these three molecules (co-lateral effects), and (iii) to analyze the genetic basis underlying adaptation to all these molecules.

## 2. Materials and Methods

**Bacterial strains.** Three wild-type clinical isolates (R192, R1435 and R1437) from the collection of the Swiss National Center for Emerging Antibiotic Resistance (NARA) and one (SB4021) from the Institut Pasteur collection were used for this study. These isolates belong to the four enterobacterial species of clinical interest, namely *K. oxytoca* (R192), *E. cloacae* (R1435), *E. coli* (R1437), and *K. pneumoniae* (SB4021). These wild-type phenotype isolates had been recovered from clinical samples (infections), and have been used rather than ATCC reference strains in order to investigate strains being as close as possible to clinical concerns.

**In-vitro experimental evolution of bacterial isolates of four species.** Evolution experiments were conducted separately for each isolate, in a duplicate manner (labeled a and b, respectively) in Mueller-Hinton (MH) liquid medium supplemented with increasing concentrations of OCT, CHX or COL used as selective agents, respectively. Minimal inhibitory concentrations (MICs) had been first determined for COL, OCT, and CHX for each wild-type strain, the latter being named ancestors thereafter. Then, for each lineage and each selective medium, 2 × 10^8^ colony forming units (CFU) obtained from an overnight culture of the ancestor were inoculated in two separate tubes in 2 mL of MH, one containing a concentration (c) equal to half the MIC value (MIC/2) and the other c equal to the MIC of the corresponding selective molecule. The latter inoculated tubes were then placed under agitation at 37 °C for 48 h. After 48 h, considered as time 1 (T1), the tubes were visually inspected and for those tubes with the higher antibiotic/antiseptic c for which a positive culture was observed, 2 × 10^8^ CFU were sampled and labeled cT1. Subsequent steps consisted of inoculating these 2 × 10^8^ CFU in 2 mL of MH containing respective antibiotic/antiseptic concentrations c, corresponding to half the MIC value (T1/2) or the MIC value (T1) of the corresponding selective molecule for the previously selected strain. For each lineage, these steps were repeated every 48 h until 10 passages were obtained, by increasing concentrations of the COL, OCT or CHX.

### 2.1. Sequencing and Analysis

#### 2.1.1. High Throughput Sequencing

Strains selected for sequencing were plated on Uriselect 4 media (Bio-Rad). One colony per Uriselect plate was then randomly picked and inoculated in LB overnight. Total bacterial DNA from that culture was extracted using QIAamp^®^ DNA Mini Kit (Qiagen, Garstligweg, Switzerland). Overall, a total of 18 lineages and 4 ancestor genomes were sequenced using the Illumina platform MiniSeq^®^ (Illumina, Cambridge, UK).

#### 2.1.2. Read Mapping and Mutations Identification

The whole genome sequences of each ancestor strain were used as respective references. Breseq 0.23 software [13] was used to identify differences between sequenced evolved genomes from the respective ancestors’ reference genomes. The pipeline breseq 0.23 uses bowtie2 [14] to map read to a reference genome. It then identifies mutations that can take the form of either read alignment (RA) evidence, corresponding to single nucleotide polymorphisms and short indels, to missing coverage, or to new junction evidence. The latter actually correspond to reads mapping to one part of the reference on one side, and to another part on the other side, indicating a possible re-arrangement. The program then uses this evidence to make mutation predictions. The RA are subsequently transformed into predictions as single-nucleotide polymorphisms (SNPs) or short indels when they are supported by at least 85% of the read. The breseq 0.23 pipeline can also identify a large deletion and chromosomal re-arrangement by possibly correlating a missing coverage in a region with a new junction evidenced on both sides of this region.

The identified mutations were filtered by breseq 0.23 [13]. When filtering the outputs of breseq 0.23, we first looked at the predicted SNPs and short indels. Mutations that seemed to appear in each sample were actually removed, considering them likely originated from a sequencing error in the reference genome and hence not informative on the dynamics of the diversification during this experiment. Mutations that were too close from one another were also removed, by discarding variations being less than 51 base pairs (bp) apart. Indeed, these clustered mutations are usually caused by reads erroneously mapped, and previous analyses showed that they are typically found in prophagic regions [15]. These mobile regions are repeated in the genome but do not have 100% identity, generating difficulties with respect to the mapping process, as they are still close enough from one another to be erroneously mapped. For instance, phage-mediated exchanges of DNA sequences among bacteria is known to occur with high frequency, resulting in constant modifications of specific regions of the genome. Finally, all mutations for which the frequency of the mutated reads was less than 0.95 were also removed, being considered as not reliable.

### 2.2. Susceptibility Testing

#### 2.2.1. Reference Antimicrobial Susceptibility Testing of the End Point Isolates

For each lineage, a single individual colony was sampled at the end of the experiment (T10), for which antimicrobial susceptibilities to a series of antibiotics were evaluated and interpreted according to the EUCAST guidelines [16]. Determination of the MICs of COL was also performed by the broth microdilution method (BMD) and interpreted according to the EUCAST/CLSI joined guidelines (http://www.eucast.org/fileadmin/src/media/PDFs/EUCAST_files/General_documents/Recommendations_for_MIC_determination_of_colistin_March_2016.pdf, accessed on 20 December 2021). Finally, the BMD method was also used to determine MICs of OCT and CHX using concentrations of each antiseptic ranging from 0.15 µg/mL to 160 µg/mL. No interpretation (categorization into susceptible/resistant) of the MIC values could be performed since no breakpoint exists for those antiseptics.

All determinations of antimicrobial susceptibilities and MICs were performed in triplicate.

#### 2.2.2. Analysis of the Results

For each lineage, the logarithm of the ratio between antibiotic (ATB) or antiseptic (ATS) MIC at the end point compared to that of the ancestor was calculated for simplification of data representation. Consequently, high/low values mean respectively that a strong/weak increase in MIC of ATB or ATS was observed between the evolved line and the ancestor.

## 3. Results and Discussion

**Figure 1:**
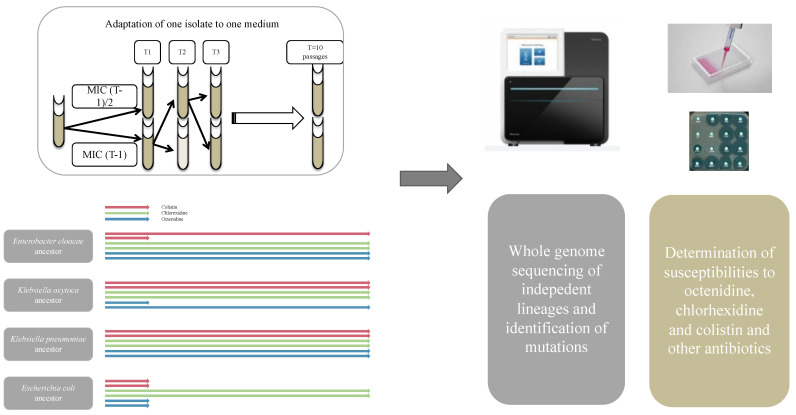
Description of the method used in the present study to obtain mutation and susceptibility data of the different lineages obtained after adaptation of 4 enterobacterial species in 3 selective media (OCT, CHX or COL). The arrows show how a selective process was conducted overtime, with some lineages ending up prematurely due to lack of selection of resistant mutants, and other lineages reaching elevated concentrations.

A total of 18 lineages could be obtained for all selective agents (OCT, CHX or COL) and for the four enterobacterial species (Table 1 and Figure 1). For four lineages, we were not able to obtain mutants adapted with selective molecules (Figure 1). Whole genome sequencing was then used in order to perform a thorough comparison between genotypes and phenotypes for all adaptive processes performed on three selective media. Mutations were actually observed in 18 lineages (Table 1), and mutants obtained at the final point of the lineage were retained for subsequent molecular analysis.

### 3.1. Adaptation on COL

Selection of mutants using COL as selective molecule enabled selection of mutants with high MIC values for *K. pneumoniae*, *K. oxytoca* and *E. cloacae*, but surprisingly not for *E. coli*. Two evolution lineages could be obtained for both *K. pneumoniae* and *K. oxytoca*, and a single one for *E. cloacae.* Out of the identified mutations, eight corresponded to non-synonymous point mutations within specific genes and one mutant possessed a mutation in an intergenic region (Table 1). 

**Figure 2:**
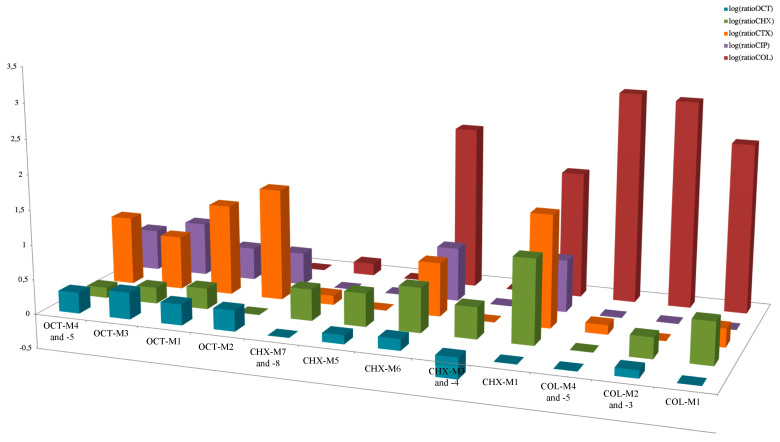
Representation of ratios of each antimicrobial MIC of each lineage divided by the corresponding MIC of the ancestor. The Ratios are represented in logarithmic scale. The mean of the ratios of the MICs of different lineages compared to the ancestor was calculated when these MICs were not significantly different and for lineages in the same media and issued from the same ancestor.

In *K. pneumoniae* and *K. oxytoca*, mutations were identified in the *lptD* gene encoding an assembly protein of the lipopolysaccharide (LPS), allowing connection between the LPS transporter to the outer membrane [17]. In those two species, mutations in two component system genes, namely *phoQ* or *rstB*, were also identified. The PhoQ protein is a sensor belonging to the two-component system regulating the biosynthesis level of LPS in many Gram-negative bacteria, and previously found to be involved in acquired resistance to polymyxins [18,19,20]. The identification of mutations in genes encoding proteins involved in LPS biosynthesis was somehow expected, considering that LPS is the main target of colistin COL action [1]. The *rstB* gene also encodes a sensor protein being part of a two-component system, the latter regulating both motility and pathogenesis of Gram-negative bacteria [21,22]. Even though the corresponding protein had never been precisely identified as being involved in resistance to polymyxins, the overexpression of the *rstB* gene was recently evidenced in a polymyxin-heteroresistant *K. pneumoniae* population [23]. Notably, two of those COL-resistant mutants showed a moderate decreased susceptibility to CHX, although OCT susceptibility remained unchanged (Figure 2, Table 2 and Table S1). This suggests that LPS modification induced by selective pressure with COL might have a collateral effect on CHX antibacterial activity, while OCT activity might be preserved in those latter cases.

In *K. oxytoca,* a single amino acid mutation was identified in the *tkt* gene encoding a putative transketolase protein of a lineage. That enzyme interacts with RamR, a key regulator involved in many regulatory processes in *E. coli.*

In *E. cloacae*, the only mutation was identified within an intergenic region, upstream of a gene encoding a YebO-like protein, belonging to a family of proteins of unknown functions being widely but exclusively distributed in *Enterobacterales* [24].

### 3.2. Adaptation on CHX

Selection of mutants using CHX as selective molecule permitted recovery of eight mutants with high MICs for all species and for each duplicate (Figure 1). Out of the identified mutations, ten corresponded to non-synonymous mutations within specific genes and four mutants possessed a mutation in an intergenic region (Table 1). In *K. pneumoniae*, the *smvA* gene encoding an efflux pump protein was found to be a target of adaptation under CHX selective pressures. This SmvA protein was previously identified as an important efflux pump for cationic biocides, belonging to the major facilitator family (MFS) and known to be involved in methyl viologen efflux [25]. Here we identified a single bp substitution in the intergenic region separating the *smvR* regulatory gene and the *smvA* gene, likely enhancing the expression of the latter by interfering with the negative regulation process.

In addition, mutations or deletions in the *malT2* gene encoding the HTH-type transcriptional activator of the *K. pneumoniae* maltose operon were identified [26]. Notably, the *K. pneumoniae* maltose operon and its MalT regulator significantly resemble those of *E. coli* [26], but no link with the regulation of these operons and adaptation to selective molecules (such as antiseptics or others) had been reported so far. 

In *E. cloacae*, a convergence of mutations within lineages exclusively obtained with CHX was observed in membrane biogenesis encoding genes (*bamE* or *mipA)*. The *bamE* encodes a putative outer membrane lipoprotein, being part of the ß-barrel assembly machine complex. Interestingly, a BamE homologous protein in *E. coli* was found to be involved in resistance to the antiparasitic drug nitazoxanide [27]. Moreover, several mutations were localized in other *tetR*-like regulatory genes, namely in *bm3R1-1* and *betI.* Interestingly, and in line with these features, all the lineages presenting mutations in the *tetR*-like regulatory genes showed concomitant decreased susceptibilities to β-lactams, quinolones, and sulfamethoxazole. By contrast, no significant modification was observed in term of susceptibility to COL, with MIC values remaining identical (Figure 2, Table 2 and Table S1). Notably, the mutants selected on CHX and exhibiting substitutions in the *bamE* and *mipA* genes showed a cross resistance to COL obtained on CHX media whereas OCT susceptibility remained unchanged (Figure 2, Table 2 and Table S1).

### 3.3. Adaptation on OCT

Selection of mutants using OCT as selective molecule permitted recovery of *K. pneumoniae, K. oxytoca* (only a single lineage), and *E. cloacae* mutants, showing for all of these species only a very slight increase (1-fold) in term of MICs. No mutant could be obtained for *E. coli*. Accordingly, an extinction was observed for three out of the eight conducted lineages with OCT as selector for the non-*E. coli* species. It was thus hypothesized that adaptation to OCT selective pressure was costly in term of fitness for those different lineages.

Nevertheless, ten mutations were observed in the five lineages showing increased MIC of OCT, of which seven actually corresponded to non-synonymous point mutations (2 stop codons), two to insertions and one to a deletion within some specific genes. The last mutant possessed an intergenic mutation (Table 1). 

Of interest, in *K. pneumoniae,* several mutations were localized in *tetR* regulatory genes (*bm3R1-1*, *betI* and *ramR)* or efflux pumps genes (*smvA*), as observed under CHX selection. Specifically, the *ramR* gene encodes a protein involved in the expression of the *acrAB-tolC* efflux system, which is well known as being involved in antimicrobial resistance in different enterobacterial species when overexpressed [28,29,30]. Indeed, all the lineages with these mutations presented decreased susceptibilities to β-lactams, quinolones, and sulfamethoxazole, whereas no significant cross-increased MICs of COL was observed (Figure 2, Table 2 and Table S1). Interestingly, Garrat et al. [31] observed similar convergence of mutations in *smvA* and *ramR* in different Gram-negative species exposed to OCT, leading to decreased susceptibility to antibiotics.

Interestingly, one of the *E. cloacae* lineages adapted to OCT showed a mutation in the *ompX* gene encoding an outer membrane protein. Most of these mutants concomitantly developed decreased susceptibilities to other antibiotics such as cefotaxime, which is in line with previous findings showing that mutations in OmpX in *E. cloacae* were basically involved in resistance to β-lactams [32]. In addition, a co-lateral effect was also noticed for ciprofloxacin for this OmpX mutant. This observation also correlates previous findings showing that OmpX is involved in the penetration not only of ß-lactams but also of fluoroquinolones in *E. cloacae* [33]. 

## 4. Conclusions

Selection of mutants showing decreased susceptibility to CHX and COL may occur for all *E. coli*, *K. pneumoniae*, *K. oxytoca* and *E. cloacae* species, leading to cross-decreased susceptibility to COL and CHX, respectively. Notably, no effect on OCT efficacy was observed for these mutants, highlighting a lack of collateral effect on that molecule. Attempts to select mutants with OCT remained difficult, and only a very limited decreased susceptibility (only one-fold) to it could be obtained for *K. pneumoniae*, *K. oxytoca* and *E. cloacae*—with cross-decreased susceptibilities to other antibiotics especially in *K. pneumoniae* (β-lactams, quinolones and sulfamethoxazole)—but no cross-effect on CHX and COL being observed. Altogether, those observations suggest that the mechanisms leading to decreased susceptibility to either of these antiseptic do not affect the other one.

Different targets of adaptation could be observed in membrane-associated genes according to the molecule used for selection. CHX adaptation lineages presented mutations in membrane biogenesis genes such as *bamE* or *mipA*, whereas OCT induced mutations in the TetR regulatory genes controlling efflux pump genes. Finally, COL adaptation was mainly related to mutations in LPS assembly associated genes or sensor proteins that did not significantly affect the susceptibility to other antibiotics such as CTX or CIP.

Analysis of the identified mutations showed a significant convergence within lineages obtained from one or both antiseptic-containing media. Overall, we have observed that mutants were very difficult to obtain with *E. coli* compared to other species. Moreover, selection of mutants with reduced susceptibility to OCT was very difficult to obtain, being infrequent and never obtained at concentrations corresponding to the ones used in clinical settings. In contrast, selection of mutants was frequent for the two other drugs tested, with numerous and worrying co-lateral effects with regard to the susceptibility to other drugs.

One limitation of our study is that the involvement of all mutations identified remains to be further confirmed by performing knock-out experiments on wild-type strains, which was beyond the primary objectives of our work.

Future work will be performed with the same approach, on other clinically-relevant bacterial species including *Pseudomonas aeruginosa* and *Acinetobacter baumannii*, since on the one hand these species are often involved in nosocomial outbreaks against which antiseptics are critical tools, and on the other hand, they are species against which colistin may be considered as a last-resort antibiotic treatment (when dealing with multidrug-resistant isolates).

## Figures and Tables

**Table 1:** Characteristics of the 18 mutants of Klebsiella pneumoniae (KP), K. oxytoca (KO), Enterobacter cloacae and Escherichia coli selected in COL, CHX or OCT media. The mutations in the lineages could be identified after whole ge-nome sequencing that could be obtained and comparison of each lineage genomic sequence with its ancestor using breseq (30). Proteins targeted by the mutation, and associated function are indicated. In brackets correspond the range of MIC in-creased, expressed in fold, by comparison to the parental strain.

Mutant Name	Bacterial Species	Selective Molecule	Lineage *	Target Mutation Proteins	Mutation Type	Function of the Corresponding Protein
**COL-M1**	*Enterobacter cloacae*	Colistin	a	Hypothetical proteins	intergenic	NA /Hypothetical protein 85% homologous to YebO
**COL-M2**	*Klebsiella oxytoca*	Colistin	a	PhoQ	M293R	Sensor protein
				Tkt	D174H	Transketolase
**COL-M3**	*K. oxytoca*	Colistin	b	LptD	T672P	LPS-assembly protein
				PhoQ	K354N	Sensor protein
				PhoQ	K46Q	Sensor protein
**COL-M4**	*Klebsiella pneumoniae*	Colistin	a	RstB	D122E	Sensor protein
**COL-M5**	*K. pneumoniae*	Colistin	b	RstB	P151L	Sensor protein
				LptD	T491P	LPS-assembly protein
**/**	*Escherichia coli*	Colistin	None	/	/	/
**CHX-M1**	*E. cloacae*	Chlorhexidine	a	BamE	Δ1 bp deletion	Outer membrane protein assembly factor
				MipA	Y219N	MltA interacting protein
**CHX-M2**	*E. cloacae*	Chlorhexidine	b	BamE	E84	Outer membrane protein assembly factor
				BetI	P66L	TetR/AcrR family transcriptionnal regulator family
				Asd/GlgB	intergenic	Aspartate-semialdehyde dehydrogenase/1.4-alpha-glucan branching enzyme
**CHX-M3**	*E. coli*	Chlorhexidine	a	Close dowstream region of MipA encoding gene	intergenic	MltA interacting protein
**CHX-M4**	*E. coli*	Chlorhexidine	b	YycB	K123T	Putative transporter
**CHX-M5**	*K. oxytoca*	Chlorhexidine	a	Lon	G383D	Lon protease
**CHX-M6**	*K. oxytoca*	Chlorhexidine	b	MoeA	K340M	Molybdopterin molybdenumtransferase
				Hypothetical proteins	intergenic	Stress induced protein with GsiB superfamily domain /NA
**CHX-M7**	*K. pneumoniae*	Chlorhexidine	a	Close uptream region of SmvA encoding gene	intergenic	Methyl viologen resistance protein
				MalT	E359K	HTH-type transcritionnal regulator
**CHX-M8**	*K. pneumoniae*	Chlorhexidine	b	MalT	Δ12bp deletion	HTH-type transcritionnal regulator
				WcaJ	Δ30 bp insertion	UDP-glucose: undecaprenyl-phosphate glucose-1-phosphate transferase
**OCT-M1**	*E. cloacae*	Octenidine	a	BetI	G187R	TetR/AcrR family transcriptionnal regulator family
				Hypothetical protein	G1114R	Putative membrane protein with AsmA- like domain (Hypothetical protein with 56% identity with YdhP from *Escherichia coli* K-12)
				NasD / TsgA	intergenic	Nitrite reductase [NAD(P)H]/ Transmembrane transporter
**OCT-M2**	*E. cloacae*	Octenidine	b	OmpX	L14P	Outer membrane Protein X precursor
**/**	*E. coli*	Octenidine	None	/	/	/
**OCT-M3**	*K. oxytoca*	Octenidine	a	Bm3R1	Δ1 bp insertion	TetR/AcrR family transcriptionnal regulator family
**OCT-M4**	*K. pneumoniae*	Octenidine	a	SmvA	A368T	Methyl viologen resistance protein
				RamR	Q122	TetR/AcrR family transcriptionnal regulateor family
				Hypothetical protein	W495	Putative membrane protein with AsmA- like domain (Protein with 72% identity with YdhP from Escherichia coli K-12)
**OCT_M5**	*K. pneumoniae*	Octenidine	b	SmvA	A474V	Methyl viologen resistance protein
				RamR	Δ1 bp deletion	TetR/AcrR family transcriptionnal regulateor family

* a and b correspond to distinct lineages of the evolutionary pathways as indicated in Mat and Met section.

**Table 2:** Susceptibilities to main antiseptics and antibiotics of the lineage compared to the ancestors. The colistin, chlorhexidine, octenidine, cefotaxime and ciprofloxacin minimal concentration inhibition of each lineage are presented in this table. Each MIC corresponded to the mean of 3 MICs determined for each couple of antiseptic or antibiotic/ lineage. In bracket are indicated the ratio of the MIC of the lineage divided by the corresponding MIC of the ancestor. T The mean of the ratios of the MICs of different lineages compared to the ancestor was calculated when these MICs were not significantly different and for lineages in the same media and issued from the same ancestor.

Isolate	Bacterial Species	Colistin MIC	Chlorhexidine MIC	Octenidine MIC	Cefotaxime MIC	Ciprofloxacin MIC
COL-M1	*E. cloacae*	32 (256)	40 (4)	2.5 (1)	0.023 (2)	0.012 (0.75)
CHX-M1	*E. cloacae*	8 (64)	160 (16)	2.5 (1)	0.5 (40)	0.064 (4)
CHX-M2	*E. cloacae*	8 (64)	160 (16)	2.5 (1)	0.5 (40)	0.125 (8)
OCT-M1	*E. cloacae*	0.125 (1)	20 (2)	5 (2)	0.25 (20)	0.047 (3)
OCT-M2	*E. cloacae*	0.125 (1)	20 (2)	2.5 (1)	0.25 (20)	0.047 (3)
CHX-M3	*E. coli*	0.125 (1)	2.5 (4)	1.25 (0.5)	0.032 (1)	0.008 (1)
CHX-M4	*E. coli*	0.125 (1)	1.25 (2)	1.25 (0.5)	0.032 (1)	0.008 (1)
COL-M2	*K. oxytoca*	96 (770)	20 (2)	2.5 (2)	0.016 (1)	0.012 (0.75)
COL-M3	*K. oxytoca*	128 (1024)	20 (2)	2.5 (2)	0.032 (2)	0.012 (0.75)
CHX-M5	*K. oxytoca*	0.125 (1)	20 (2)	2.5 (2)	0.016 (1)	0.016 (1)
CHX-M6	*K. oxytoca*	16 (128)	40 (4)	2.5 (2)	0.094 (6)	0.094 (6)
OCT-M3	*K. oxytoca*	0.125 (1)	20 (2)	5 (4)	0.094 (6)	0.094 (6)
COL-M4	*K. pneumoniae*	256 (1024)	10 (1)	1.25 (1)	0.032 (1)	0.016 (0.5)
COL-M5	*K. pneumoniae*	256 (1024)	10 (1)	1.25 (1)	0.032 (1)	0.016 (0.5)
CHX-M7	*K. pneumoniae*	0.25 (1)	40 (4)	1.25 (1)	0.032 (1)	0.016 (0.5)
CHX-M8	*K. pneumoniae*	0.25 (1)	20 (2)	1.25 (1)	0.032 (1)	0.016 (0.5)
OCT-M4	*K. pneumoniae*	0.25 (1)	10 (1)	2.5 (2)	0.25 (8)	0.125 (4)
OCT-M5	*K. pneumoniae*	0.25 (1)	20 (2)	2.5 (2)	0.38 (12)	0.125 (4)

## Data Availability

Data can be obtained upon request to laurent.poirel@unifr.ch.

## Supplementary Materials

The following supporting information can be downloaded at: https://www.mdpi.com/article/10.3390/antibiotics11010050/s1, Table S1: Genotypic and phenotypic characteristics of the mutants obtained after adaptation on OCT, chlorhexidine or colistin of four enterobacterial species (*Klebsiella pneumoniae* (KP), *K. oxytoca* (KO)*, Enterobacter cloacae* and *Escherichia coli*) and their ancestors.
