# Supplementary material for: Co-Lateral Effect of Octenidine, Chlorhexidine and Colistin Selective Pressures on Four Enterobacterial Species: A Comparative Genomic Analysis
Source: Antibiotics (Basel). 2021 Dec 31;11(1):50. doi: 10.3390/antibiotics11010050 (PMC8772718; doi:10.3390/antibiotics11010050)
Supplement: Supplementary file 1 [file antibiotics-11-00050-s001.zip › antibiotics-1450369-supplementary.pdf]

Isolate	*Bacterial species*	Mutation targeted gene	Associated protein information	Mutation	Mutation type	Colistin MIC	Chlorhexidine MIC	Octenidine MIC	Cefotaxime MIC	Ciprofloxacin MIC	AMX	AMXCLAV	TIC	TICCLAV	PIPTAZ	PIP	TEM	FOX	CTX	AZT	CAZ	FEP	IMP	ERT	GEN	NET	TOB	AMK	NAL	OFX	CIP	SXT	FOS
Enterobacter cloacae wild type	*E. cloacae*	/	/	/	/	0.125	10	2.5	0.0125	0.016	9	9	30	33	28	26	31	7	29	38	28	35	31	32	22	28	23	24	25	35	40	30	21
COL-M1	*E. cloacae*	– / → Gene_01288	NA /Hypothetical protein 85% homologous to YebO	G→T	intergenic	32	40	2.5	0.023	0.012	8	8	26	26	24	23	25	6	24	34	24	30	24	27	23	23	21	22	24	33	38	28	19
CHX-M1	*E. cloacae*	*bamE* ←	Outer membrane protein assembly factor BamE	Δ1 bp	deletion	8	160	2.5	0.5	0.064	8	8	27	27	19	24	20	6	23	37	26	29	29	31	26	29	26	26	23	31	38	28	20
		mipA →	MltA interacting protein	T→A	Y219N																												
CHX-M2	*E. cloacae*	*bamE* ←	Outer membrane protein assembly factor BamE	A→C	*84E	8	160	2.5	0.5	0.125	8	8	26	28	20	23	22	6	24	36	27	32	28	28	26	30	26	25	22	31	37	26	20
		betI →	TetR/AcrR family transcriptionnal regulator family	C→T	P66L																												
		asd → / → *glgB*	Aspartate-semialdehyde dehydrogenase/1.4-alpha-glucan branching enzyme GlgB	G→T	intergenic																												
OCT-M1	*E. cloacae*	*betI* →	TetR/AcrR family transcriptionnal regulateor family	G→A	G187R	0.125	20	5.0	0.25	0.047	6	6	29	28	24	24	23	10	27	38	29	34	30	35	25	29	26	26	15	30	38	24	21
		Gene _02347 →	Putative membrane protein with AsmA- like domain. (56% identity with YdhP from Escherichia coli K-12)	G→A	G1114R																												
		nasD ← / ← *tsgA*	Nitrite reductase [NAD(P)H]/ transmembrane transporter	C→A	intergenic																												
OCT-M2	*E. cloacae*	*ompX*	Outer membrane Protein X precursor	A→G	L14P	0.125	20	2.5	0.25	0.047	6	6	25	25	21	21	23	6	24	35	26	34	30	35	25	25	24	25	15	28	31	25	20
Escherichia coli wt	*E. coli*	/	/	/	/	0.125	0.625	2.5	0.032	0.008	25	27	30	35	29	27	23	27	36	40	33	38	37	37	27	29	26	29	25	39	40	32	33
CHX-M3	*E. coli*	*mipA* →	MltA interacting protein	A→G	intergenic	0.125	2.5	1.25	0.032	0.008	24	30	29	34	27	23	21	29	38	40	32	38	37	31	28	29	27	29	32	40	40	40	31
CHX-M4	*E. coli*	*yycB* →	putative transporter YycB	A→C	K123T	<0.1	1.25	1.25	0.032	0.0016	24	28	31	34	29	29	22	29	35	40	32	38	32	40	31	40	36	40	31	40	40	40	29
Klebsiella oxytoca wt	*K. oxytoca*	/	/	/	/	0.125	9	1.7	0.016	0.016	6	34	13	35	31	19	30	32	37	40	36	39	40	40	28	26	29	30	29	37	40	34	21
COL-M2	*K. oxytoca*	*phoQ* ←	Sensor protein PhoQ	A→C	M293R	96	20	2.5	0.016	0.012	6	29	6	30	20	17	26	22	33	38	30	37	36	28	26	30	29	29	22	32	39	30	21
		tkt ←	Transketolase	C→G	D174H																												
COL-M3	*K. oxytoca*	*lptD* ←	LPS-assembly protein LptD	T→G	T672P	128	16.7	2.0	0.032	0.012	6	36	6	40	29	19	29	36	36	40	37	38	36	38	26	27	27	23	30	40	40	32	22
		phoQ ←	Sensor protein PhoQ	C→A	K354N																												
		phoQ ←	Sensor protein PhoQ	T→G	K46Q																												
CHX-M5	*K. oxytoca*	*lon_1* ←	Lon protease	C→T	G383D	0.125	27.5	2.3	0.016	0.016	6	36	6	40	29	19	29	36	36	40	37	38	36	38	26	27	27	23	30	40	40	32	22
CHX-M6	*K. oxytoca*	*moeA* ←	Molybdopterin molybdenumtransferas	T→A	K340M	26.7	40	2.5	0.094	0.094	6	35	6	33	30	19	30	30	37	40	35	38	40	37	29	28	29	31	31	38	40	40	22
		Gene_3346 → / –	Stress induced protein with GsiB superfamily domain / –	C→A	intergenic																												
OCT-M3	*K. oxytoca*	*bm3R1_1*	TetR/AcrR family transcriptionnal regulateor family	+ C	insertion	0.125	16	4.2	0.094	0.094	6	35	6	33	30	19	30	30	37	40	35	38	40	37	29	28	29	31	31	38	40	40	22
Klebsiella pneumoniae wt	*K. pneumoniae*	/	/	/	/	0.25	10	1.25	0.032	0.032	6	29	10	30	26	19	28	27	33	37	31	35	33	34	28	30	25	25	25	35	40	36	18
COL-M4	*K. pneumoniae*	*rstB_2* →	Sensor protein RstB	C→A	D122E	256	10	1.25	0.032	0.016	6	27	6	24	19	18	18	14	30	40	26	32	38	36	29	31	29	29	18	28	36	26	22
COL-M5	*K. pneumoniae*	*rstB_2* →	Sensor protein RstB	C→T	P151L	256	10	1.25	0.032	0.016	6	25	6	23	16	16	15	16	30	37	24	33	37	36	28	30	28	28	17	26	34	24	20
		lptD ←	LPS-assembly protein LptD	T→G	T491P																												
CHX-M7	*K. pneumoniae*	*smvA* ←	Methyl viologen resistance protein SmvA	C→A	intergenic	0.25	40	1.25	0.032	0.016	6	29	10	30	26	19	28	27	33	37	31	35	33	34	28	30	25	25	25	35	40	36	18
		malT_2 →	HTH-type transcritionnal regulator MalT	G→A	E359K																												
CHX-M8	*K. pneumoniae*	*malT_2* →	HTH-type transcritionnal regulator MalT	Δ12bp	deletion	0.25	20	1.25	0.032	0.016	6	29	10	30	26	19	28	27	33	37	31	35	33	34	28	30	25	25	25	35	40	36	18
		wcaJ ←	UDP-glucose: undecaprenyl-phosphate glucose-1-phosphate transferase	(A)5 → 6	insertion																												
OCT-M4	*K. pneumoniae*	*smvA* ←	Methyl viologen resistance protein SmvA	C→T	A368T	0.25	10	2.5	0.25	0.125	6	29	10	30	26	19	28	27	33	37	31	35	33	34	28	30	25	25	25	35	40	36	18
		ramR ←	TetR/AcrR family transcriptionnal regulateor family	G→A	Q122*																												
		Gene_04779 ←	Putative membrane protein with AsmA- like domain. (72% identity with YdhP from Escherichia coli K-12)	C→T	W495*																												
OCT-M5	*K. pneumoniae*	*smvA* ←	Methyl viologen resistance protein SmvA	G→A	A474V	0.25	20	2.5	0.38	0.125	6	29	10	30	26	19	28	27	33	37	31	35	33	34	28	30	25	25	25	35	40	36	18
		ramR ←	TetR/AcrR family transcriptionnal regulateor family	Δ1bp	deletion																												
